# Supplementary material for: In Silico Analysis of Bioactive Peptides Released from Giant Grouper (Epinephelus lanceolatus) Roe Proteins Identified by Proteomics Approach
Source: Molecules. 2018 Nov 8;23(11):2910. doi: 10.3390/molecules23112910 (PMC6278403; doi:10.3390/molecules23112910)
Supplement: Supplementary file 1 [file molecules-23-02910-s001.pdf]

In silico **analysis of bioactive peptides released from giant grouper (*Epinephelus lanceolatus*) roe proteins identified by proteomics approach**

Fenny Crista A. Panjaitan [1], Honey Lyn R. Gomez [2], Yu-Wei Chang [1,*]

[1] *Department of Food Science, National Taiwan Ocean University, Keelung 202, Taiwan*

[2] Institute of Fish Processing Technology, College of Fisheries and Ocean Sciences, University of the Philippines Visayas, Miagao 5023, Iloilo, Philippines

**Corresponding author*

Supplementary Figure S1

Figure S1. Amino acid sequences of vitellogenin from giant grouper (*Epinephelus lanceolatus*)[1].

MKAVV	LALTL	AFVAG	QNFAP	EFAAG	KTYVY	KYEAL	ILGGL	PEEGL	ARAGL	50
KISTK	LLLSA	ADQNT	YMLKL	VEPEL	SEYSG	IWPKD	PAVPA	TKLTA	ALAPQ	100
LAIPI	KFEYT	NGVVG	KVFAP	EEVST	LVLNI	YRGIL	NILQL	NIKKT	HKVYD	150
LQEVG	TQGVC	KTLYS	ISEDA	RIENI	LLTKT	RDLSN	CQERL	NKDIG	LAYTE	200
KCDKC	QEETK	NLRGT	TTLSY	VLKPV	ADAVM	ILKAY	VNELI	QFSPF	SEANG	250
AAQMR	TKQSL	EFLEI	EKEPI	PSVKA	EYRHR	GSLKY	EFSDE	LLQTP	LQLIK	300
ISDAP	AQVAE	VLKHL	ATYNI	EDVHE	NAPLK	FLELV	QLLRI	ARYED	LEMYW	350
NQYKK	MSPHR	HWFLD	TIPAT	GTFAG	LRFIK	EKFMA	EEITI	AEAAQ	AFITA	400
VHMVT	ADPEV	AKLFE	SLVDS	DKVVE	NPLLR	EVVFL	GYGTM	VNKYC	NKTVD	450
CPVEL	IKPIQ	QRLSD	AIAKN	EEENI	ILYIK	VLGNA	GHPSS	FKSLT	KIMPI	500
HGTAA	VSLPM	TIHVE	AIMAL	RNIAK	KESRM	VQELA	LQLYM	DKALH	PELRM	550
LSCIV	LFETS	PSMGL	VTTVA	NSVKT	EENLQ	VASFT	YSHMK	SLSRS	PATIH	600
PDVAA	ACSAA	MKILG	TKLDR	LSLRY	SKAVH	VDLYN	SSLAV	GAAAT	AFYIN	650
DAATF	MPKSF	VAKTK	GFIAG	STAEV	LEIGA	NIEGL	QELIL	KNPAL	SESTD	700
RITKM	KRVIK	ALSEW	RSLPT	SKPLA	SVYVK	FFGQE	IGFAN	IDKPM	IDKAV	750
KFGKE	LFIQE	YGREA	LKALL	LSGIN	FHYAK	FVLAA	EMRRI	LPTVA	GIPME	800
LSLYS	AAVAA	ASVEI	KPNTS	PRLSA	DFDVK	TLLET	DVELK	AEIRP	MVAMD	850
TYAVM	GLNTD	IFQAA	LVARA	KLHSV	VPAKI	AARLN	IKEGD	FKLEA	LPVDV	900
PENIT	SMNVT	TFAVA	RNIEE	PLVER	ITPLL	PTKVL	VPIPI	RRHTS	KLDPT	950
RNSML	DSSEL	LPMEE	EDVEP	IPEYK	FRRFA	KKYCA	KHIGV	GLKAC	FKFAS	1000
QNGAS	IQDIV	LYKLA	GSHNF	SFSVT	PIEGE	VVERL	EMEVK	VGAKA	AEKLV	1050
KRINL	SEDEE	TEEGG	PVLVK	LKKIL	SSRRN	SSSSS	SSSSS	SSSSS	ESRSS	1100
RSSSS	SSSSS	SSSSR	KIDLA	ARTNS	SSSSS	SSRNS	SSSSS	SSSSS	SSSSS	1150
SSSSS	SSSSR	RSSSS	SSSSS	SSSSS	SSSSR	RVNST	RSSSS	SSRTS	SASSL	1200
ASFFS	DSSSS	SSSSD	RRSKE	VMEKF	QRLHK	KMVAS	GSSAS	SVNEA	IYKEK	1250
KYLGE	EAVVA	VILRA	VKADK	RMVGY	QLGFY	LDKPN	ARVQI	IVANI	SSSDS	1300
NWRIC	ADAVV	LSKHK	VTTKI	SWGEQ	CRKYS	TNVTG	ETGIV	SSSPA	ARLRV	1350
SWERL	PSTLK	RYGKM	VNKYV	PVKIL	SDLIH	TKREN	STRNI	SVIAV	ATSEK	1400
TIDII	TKTPM	SSSVY	NVKYV	PVKIL	SDLIH	TKREN	STRNI	SVIAV	ATSEK	1450
AAAAE	CSFVE	DTLYT	FNNRS	YKNKM	PSSCY	QVAAQ	DCTDE	LKFMV	LLRKD	1500
SSEQH	HINVK	ISEID	IDMFP	KDDNV	TVKVN	EMEIP	PPACL	TATQQ	LPLKI	1550
KTKRR	GLAVY	APSHG	LQEVY	FDRKT	WRIKV	ADWMK	GKTCG	LCGKA	DGEIR	1600
QEYHT	PNGRV	AKNSI	SFAHS	WILPA	ESCRD	ASECR	LKLES	VQLEK	QLTIH	1650
GEDST	CFSVE	PVPRC	LPGCL	PVKTT	PVTVG	FSCLA	SDPQT	SVYDR	SVDLR	1700
QTTQA	HLACS	CNTKC	S							1716

Reference

1. Om, A.D.; Jasmani, S.; Ismail, N.; Yeong, S.Y.; Abol-Munafi, A.B. Application MALDI TOF on protein identification of vitellogenin in giant grouper (*Epinephelus lanceolatus*). *Fish Physiol. Biochem.* **2013**, *39*, 1277-1286, doi:https://doi.org/10.1007/s10695-013-9782-x.

In silico analysis of bioactive peptides released from giant grouper (_Epinephelus lanceolatus_) roe proteins identified by proteomics approach

Fenny Crista A. Panjaitan [1], Honey Lyn R. Gomez [2], Yu-Wei Chang [1,*]

[1] *Department of Food Science, National Taiwan Ocean University, Keelung 202, Taiwan*

[2] Institute of Fish Processing Technology, College of Fisheries and Ocean Sciences, University of the Philippines Visayas, Miagao 5023, Iloilo, Philippines

*Corresponding author

Supplementary Table S1

Table S1. Identified tryptic peptides of vitellogenin (originated from *Epinephelus coioides*; NCBI accession number: AAW29031.1; Protein sequence coverage: 41%; 615 aa; excised from band B; accessed on 21 February 2018) in Mascot search results.

Position Start-End	Peptide	Modification	Observed (m/z)	Mr (expt)	Mr (calc)
37 – 51	K.LVEPELFEYSGVYPK.D		885.45	1768.89	1768.89
37 – 59	K.LVEPELFEYSGVYPKDPLVPATK.L		864.46	2590.36	2590.36
74 – 83	K.FEYANGVVGK.V		542.28	1082.54	1082.54
100 – 111	R.GILNVLQLNIKK.T		676.94	1351.86	1351.86
112 – 128	K.TQNVYEMQEAGAQGVCK.T	Carbamidomethyl (C)	956.93	1911.85	1911.85
129 – 138	K.TLYAISEDEK.A		584.79	1167.57	1167.57
129 – 141	K.TLYAISEDEKAER.I		762.88	1523.75	1523.75
160 – 168	K.DMGLAYTEK.C		514.24	1026.47	1026.47
225 – 234	K.QSLVFLEIQR.A		616.85	1231.69	1231.69
235 – 247	R.APIVPIQAEYLHR.G		753.93	1505.84	1505.84
252 – 267	K.YEFSTELLQTPIQLIK.I		962.03	1922.05	1922.04
290 – 297	K.VHEDAPLK.F		454.75	907.48	907.48
298 – 306	K.FLELIQLLR.V		572.86	1143.70	1143.70
310 – 321	K.FEDLEVIWSQFK.T		770.89	1539.77	1539.76
328 – 344	R.QWILDAIPAIGTPAALK.F		889.52	1777.02	1777.01
390 – 397	K.IVENPVLR.E		470.29	938.56	938.55
390 – 410	K.IVENPVLREIVLLGYGTMISK.Y	Oxidation (M)	787.45	2359.32	2359.32
398 – 410	R.EIVLLGYGTMISK.Y		712.40	1422.78	1422.78
437 – 447	K.DETQNIILFLK.V		667.37	1332.73	1332.73
448 – 463	K.VLGNAGHPTSLKPITK.I		816.98	1631.94	1631.94
479 – 488	R.VHADAIMALR.N		548.80	1095.59	1095.59
497 – 509	R.MIQELALQLYMDK.A	Oxidation (M)	806.41	1610.81	1610.80
562 – 575	K.STTAIHASVAAACK.V	Carbamidomethyl (C)	694.35	1386.69	1386.69

In silico analysis of bioactive peptides released from giant grouper (*Epinephelus lanceolatus*) roe proteins identified by proteomics approach

Fenny Crista A. Panjaitan [1], Honey Lyn R. Gomez [2], Yu-Wei Chang [1,*]

[1] *Department of Food Science, National Taiwan Ocean University, Keelung 202, Taiwan*

[2] Institute of Fish Processing Technology, College of Fisheries and Ocean Sciences, University of the Philippines Visayas, Miagao 5023, Iloilo, Philippines

*Corresponding author

Supplementary Table S2

Table S2. List of bioactive peptides analyzed by BIOPEP database from vitellogenin (originated from *Epinephelus coioides*; NCBI accession number: AAW29031.1; accessed on 30 October 2018).

BIOPEP ID	Activity	Number of peptide	Sequence	Location
2642	ACE inhibitor	1	ILP	[464-466]
3257	ACE inhibitor	2	RL	[584-585],[587-588]
3258	ACE inhibitor	1	IR	[423-424]
3361	ACE inhibitor	1	LKL	[35-37]
3379	ACE inhibitor	1	AKK	[491-493]
3381	ACE inhibitor	2	LY	[130-131],[505-506]
3384	ACE inhibitor	2	VF	[84-85],[228-229]
3393	ACE inhibitor	1	FAP	[85-87]
3489	ACE inhibitor	1	RF	[591-592]
3492	ACE inhibitor	3	VY	[48-49],[97-98],[115-116]
3505	ACE inhibitor	1	VYP	[48-50]
3507	ACE inhibitor	1	IPA	[334-336]
3518	ACE inhibitor	1	VAA	[570-572]
3532	ACE inhibitor	1	GY	[403-404]
3537	ACE inhibitor	2	PR	[495-496],[583-584]
3539	ACE inhibitor	1	LAA	[64-66]
3541	ACE inhibitor	1	LSP	[581-583]
3550	ACE inhibitor	2	YL	[244-245],[350-351]
3551	ACE inhibitor	3	LF	[42-43],[444-445],[523-524]
3553	ACE inhibitor	1	YG	[404-405]
3556	ACE inhibitor	2	FY	[600-601],[614-615]
3558	ACE inhibitor	1	LAY	[163-165]
3563	ACE inhibitor	2	AY	[164-165],[325-326]
3597	ACE inhibitor	1	AIP	[333-335]
3666	ACE inhibitor	1	YP	[49-50]
7509	ACE inhibitor	1	GLP	[6-8]
7513	ACE inhibitor	3	PL	[53-54],[295-296],[604-605]
7544	ACE inhibitor	1	IW	[316-317]
7547	ACE inhibitor	1	IRP	[423-425]
7549	ACE inhibitor	2	LKP	[189-191],[458-460]
7558	ACE inhibitor	1	VK	[540-541]
7562	ACE inhibitor	5	IA	[357-358],[365-366],[381-382],[490-

				491],[536-537]
7581	ACE inhibitor	1	IP	[334-335]
7582	ACE inhibitor	3	RP	[323-324],[424-425],[527-528]
7583	ACE inhibitor	1	AF	[613-614]
7584	ACE inhibitor	3	AP	[86-87],[235-236],[294-295]
7585	ACE inhibitor	7	LA	[12-13],[64-65],[163-164],[351-352],[430-431],[501-502],[518-519]
7587	ACE inhibitor	3	VP	[55-56],[238-239],[368-369]
7588	ACE inhibitor	2	RA	[14-15],[234-235]
7589	ACE inhibitor	2	YA	[76-77],[131-132]
7590	ACE inhibitor	9	AA	[65-66],[218-219],[341-342],[360-361],[472-473],[571-572],[572-573],[609-610],[610-611]
7594	ACE inhibitor	2	VG	[81-82],[192-193]
7595	ACE inhibitor	1	IG	[337-338]
7596	ACE inhibitor	4	GI	[16-17],[89-90],[100-101],[195-196]
7598	ACE inhibitor	4	GA	[122-123],[181-182],[217-218],[608-609]
7599	ACE inhibitor	4	GL	[6-7],[11-12],[162-163],[531-532]
7600	ACE inhibitor	3	AG	[15-16],[121-122],[452-453]
7601	ACE inhibitor	2	GH	[193-194],[453-454]
7602	ACE inhibitor	1	HL	[281-282]
7603	ACE inhibitor	1	GR	[586-587]
7604	ACE inhibitor	1	KG	[10-11]
7606	ACE inhibitor	3	DA	[293-294],[332-333],[482-483]
7607	ACE inhibitor	1	GS	[248-249]
7608	ACE inhibitor	3	GV	[47-48],[79-80],[125-126]
7609	ACE inhibitor	2	MG	[161-162],[530-531]
7611	ACE inhibitor	1	GK	[82-83]
7612	ACE inhibitor	3	GT	[338-339],[405-406],[469-470]
7614	ACE inhibitor	2	HG	[194-195],[468-469]
7616	ACE inhibitor	1	GG	[5-6]
7617	ACE inhibitor	1	QG	[124-125]
7618	ACE inhibitor	1	SG	[46-47]
7619	ACE inhibitor	5	LG	[4-5],[402-403],[449-450],[585-586],[607-608]
7622	ACE inhibitor	1	EG	[88-89]
7623	ACE inhibitor	5	EA	[120-121],[200-201],[359-360],[376-377],[432-433]
7624	ACE inhibitor	2	NG	[78-79],[216-217]
7626	ACE inhibitor	2	IAK	[381-383],[490-492]
7682	ACE inhibitor	1	NY	[186-187]
7684	ACE inhibitor	2	SY	[184-185],[552-553]
7685	ACE inhibitor	1	SF	[550-551]
7691	ACE inhibitor	3	KY	[251-252],[349-350],[410-411]
7692	ACE inhibitor	4	KF	[73-74],[297-298],[309-310],[344-345]
7693	ACE inhibitor	4	KL	[36-37],[59-60],[379-380],[383-384]
7698	ACE inhibitor	1	NK	[388-389]
7742	ACE inhibitor	1	AR	[13-14]
7743	ACE inhibitor	3	KA	[138-139],[509-510],[594-595]
7746	ACE inhibitor	1	LVE	[37-39]
7752	ACE inhibitor	3	EY	[44-45],[75-76],[243-244]
7810	ACE inhibitor	2	KP	[190-191],[459-460]
7824	ACE inhibitor	1	IAE	[357-359]
7826	ACE inhibitor	2	EI	[231-232],[398-399]
7828	ACE inhibitor	2	EV	[277-278],[314-315]

7829	ACE inhibitor	5	VE	[38-39],[276-277],[287-288],[391-392],[420-421]
7830	ACE inhibitor	4	TE	[166-167],[256-257],[375-376],[542-543]
7831	ACE inhibitor	4	LQ	[105-106],[259-260],[503-504],[546-547]
7832	ACE inhibitor	5	LN	[95-96],[102-103],[107-108],[150-151],[279-280]
7833	ACE inhibitor	1	PT	[455-456]
7834	ACE inhibitor	2	TQ	[112-113],[439-440]
7836	ACE inhibitor	1	PP	[528-529]
7839	ACE inhibitor	1	ME	[221-222]
7840	ACE inhibitor	6	EK	[9-10],[137-138],[155-156],[167-168],[288-289],[348-349]
7841	ACE inhibitor	2	KE	[347-348],[493-494]
7842	ACE inhibitor	2	HP	[454-455],[512-513]
8193	ACE inhibitor	8	AI	[132-133],[333-334],[336-337],[377-378],[483-484],[565-566],[577-578],[595-596]
8404	ACE inhibitor	1	VLKP	[188-191]
8951	ACE inhibitor	1	AV	[433-434]
8968	ACE inhibitor	1	ASL	[473-475]
9037	ACE inhibitor	1	GKV	[82-84]
9047	ACE inhibitor	1	AQL	[66-68]
9051	ACE inhibitor	1	RPP	[527-529]
9053	ACE inhibitor	1	FYN	[600-602]
9065	ACE inhibitor	1	GVY	[47-49]
9068	ACE inhibitor	1	VTR	[283-285]
9073	ACE inhibitor	4	TP	[70-71],[261-262],[339-340],[470-471]
9074	ACE inhibitor	1	DF	[599-600]
9075	ACE inhibitor	2	DM	[160-161],[428-429]
9077	ACE inhibitor	1	YV	[187-188]
9078	ACE inhibitor	2	YE	[116-117],[252-253]
9079	ACE inhibitor	8	IL	[101-102],[142-143],[196-197],[198-199],[330-331],[443-444],[464-465],[580-581]
9173	ACE inhibitor	3	RG	[99-100],[180-181],[247-248]
9184	ACE inhibitor	3	ST	[91-92],[255-256],[562-563]
9185	ACE inhibitor	2	YN	[185-186],[601-602]
9191	ACE inhibitor	1	GHG	[193-195]
9213	ACE inhibitor	6	LR	[179-180],[305-306],[396-397],[487-488],[515-516],[590-591]
8268	antibacterial	1	YVL	[187-189]
2882	immunomodulating	1	YG	[404-405]
3356	stimulating	1	LLL	[2-4]
8320	stimulating	9	VL	[23-24],[94-95],[104-105],[188-189],[278-279],[395-396],[400-401],[448-449],[522-523]
8321	stimulating	5	LV	[37-38],[54-55],[227-228],[282-283],[532-533]
8322	stimulating	6	IV	[237-238],[275-276],[390-391],[399-400],[521-522],[539-540]
8323	stimulating	8	IL	[101-102],[142-143],[196-197],[198-199],[330-331],[443-444],[464-465],[580-581]
8324	stimulating	9	LI	[24-25],[197-198],[206-207],[265-266],[301-302],[364-365],[380-381],[384-

				385],[422-423]
8325	stimulating	1	II	[442-443]
8326	stimulating	7	LL	[2-3],[3-4],[68-69],[143-144],[258-259],[304-305],[401-402]
8330	stimulating	1	SE	[134-135]
3099	immunostimulating	1	LGY	[402-404]
8310	neuropeptide	2	YL	[244-245],[350-351]
2738	regulating	1	LGY	[402-404]
3305	antioxidative	2	LH	[245-246],[511-512]
3317	antioxidative	1	HL	[281-282]
7862	antioxidative	1	IKK	[109-111]
7866	antioxidative	2	AY	[164-165],[325-326]
7868	antioxidative	1	ADF	[598-600]
7872	antioxidative	2	LY	[130-131],[505-506]
7888	antioxidative	8	EL	[41-42],[205-206],[257-258],[300-301],[354-355],[421-422],[500-501],[514-515]
7968	antioxidative	1	YNY	[185-187]
7999	antioxidative	1	LHR	[245-247]
8107	antioxidative	1	IKL	[378-380]
8132	antioxidative	1	KAI	[594-596]
8133	antioxidative	1	KVI	[156-158]
8134	antioxidative	3	KD	[51-52],[159-160],[436-437]
8139	antioxidative	2	PEL	[40-42],[513-515]
8150	antioxidative	1	YVL	[187-189]
8215	antioxidative	1	IR	[423-424]
8216	antioxidative	2	LKP	[189-191],[458-460]
8217	antioxidative	7	LK	[35-36],[189-190],[250-251],[296-297],[343-344],[446-447],[458-459]
8218	antioxidative	2	KP	[190-191],[459-460]
8224	antioxidative	3	VY	[48-49],[97-98],[115-116]
8484	antioxidative	1	LLR	[304-306]
8983	antioxidative	2	GAA	[217-219],[608-610]
9355	antioxidative	1	LAC	[518-520]
9360	antioxidative	1	LPM	[475-477]
9366	antioxidative	1	KYL	[349-351]
3751	bacterial permease ligand	2	KK	[110-111],[492-493]
8247	inhibitor	1	IR	[423-424]
8249	inhibitor	4	KF	[73-74],[297-298],[309-310],[344-345]
8250	inhibitor	1	EF	[253-254]
8246	hypotensive	1	IR	[423-424]
8248	hypotensive	4	KF	[73-74],[297-298],[309-310],[344-345]
8251	hypotensive	1	EF	[253-254]
4005	activating ubiquitin-mediated proteolysis	2	RA	[14-15],[234-235]
4006	activating ubiquitin-mediated proteolysis	7	LA	[12-13],[64-65],[163-164],[351-352],[430-431],[501-502],[518-519]
3170	dipeptidyl peptidase IV inhibitor	1	PP	[528-529]
3171	dipeptidyl peptidase IV inhibitor	1	MP	[415-416]
3172	dipeptidyl peptidase IV inhibitor	5	VA	[307-308],[434-435],[548-549],[570-571],[576-577]
3173	dipeptidyl peptidase IV	1	MA	[485-486]

	inhibitor			
3174	dipeptidyl peptidase IV inhibitor	3	KA	[138-139],[509-510],[594-595]
3175	dipeptidyl peptidase IV inhibitor	7	LA	[12-13],[64-65],[163-164],[351-352],[430-431],[501-502],[518-519]
3176	dipeptidyl peptidase IV inhibitor	2	FA	[85-86],[212-213]
3177	dipeptidyl peptidase IV inhibitor	3	AP	[86-87],[235-236],[294-295]
3179	dipeptidyl peptidase IV inhibitor	5	PA	[56-57],[324-325],[335-336],[340-341],[471-472]
3180	dipeptidyl peptidase IV inhibitor	3	LP	[7-8],[465-466],[475-476]
3181	dipeptidyl peptidase IV inhibitor	3	VP	[55-56],[238-239],[368-369]
3182	dipeptidyl peptidase IV inhibitor	7	LL	[2-3],[3-4],[68-69],[143-144],[258-259],[304-305],[401-402]
3183	dipeptidyl peptidase IV inhibitor	1	VV	[80-81]
3184	dipeptidyl peptidase IV inhibitor	3	HA	[480-481],[567-568],[597-598]
8304	dipeptidyl peptidase IV inhibitor	1	IPA	[334-336]
8501	dipeptidyl peptidase IV inhibitor	1	IP	[334-335]
8503	dipeptidyl peptidase IV inhibitor	4	TP	[70-71],[261-262],[339-340],[470-471]
8505	dipeptidyl peptidase IV inhibitor	2	SP	[210-211],[582-583]
8518	dipeptidyl peptidase IV inhibitor	3	RP	[323-324],[424-425],[527-528]
8519	dipeptidyl peptidase IV inhibitor	2	KP	[190-191],[459-460]
8520	dipeptidyl peptidase IV inhibitor	2	HP	[454-455],[512-513]
8521	dipeptidyl peptidase IV inhibitor	1	YP	[49-50]
8524	dipeptidyl peptidase IV inhibitor	4	GA	[122-123],[181-182],[217-218],[608-609]
8525	dipeptidyl peptidase IV inhibitor	5	IA	[357-358],[365-366],[381-382],[490-491],[536-537]
8526	dipeptidyl peptidase IV inhibitor	2	RA	[14-15],[234-235]
8529	dipeptidyl peptidase IV inhibitor	2	EP	[39-40],[494-495]
8530	dipeptidyl peptidase IV inhibitor	2	NP	[393-394],[603-604]
8531	dipeptidyl peptidase IV inhibitor	4	TA	[28-29],[62-63],[372-373],[564-565]
8555	dipeptidyl peptidase IV inhibitor	3	FL	[229-230],[298-299],[445-446]
8557	dipeptidyl peptidase IV inhibitor	1	HL	[281-282]
8558	dipeptidyl peptidase IV inhibitor	6	EK	[9-10],[137-138],[155-156],[167-168],[288-289],[348-349]
8559	dipeptidyl peptidase IV	6	AL	[63-64],[342-343],[363-364],[486-

	inhibitor			487],[502-503],[510-511]
8560	dipeptidyl peptidase IV inhibitor	6	SL	[226-227],[249-250],[457-458],[474-475],[558-559],[589-590]
8561	dipeptidyl peptidase IV inhibitor	4	GL	[6-7],[11-12],[162-163],[531-532]
8637	dipeptidyl peptidase IV inhibitor	9	AA	[65-66],[218-219],[341-342],[360-361],[472-473],[571-572],[572-573],[609-610],[610-611]
8638	dipeptidyl peptidase IV inhibitor	3	PL	[53-54],[295-296],[604-605]
8679	dipeptidyl peptidase IV inhibitor	1	WI	[329-330]
8687	dipeptidyl peptidase IV inhibitor	1	WS	[317-318]
8696	dipeptidyl peptidase IV inhibitor	1	YT	[165-166]
8757	dipeptidyl peptidase IV inhibitor	3	AD	[352-353],[481-482],[598-599]
8758	dipeptidyl peptidase IV inhibitor	7	AE	[29-30],[139-140],[213-214],[242-243],[358-359],[413-414],[431-432]
8759	dipeptidyl peptidase IV inhibitor	1	AF	[613-614]
8760	dipeptidyl peptidase IV inhibitor	3	AG	[15-16],[121-122],[452-453]
8762	dipeptidyl peptidase IV inhibitor	5	AS	[366-367],[473-474],[549-550],[568-569],[611-612]
8763	dipeptidyl peptidase IV inhibitor	4	AT	[27-28],[57-58],[182-183],[201-202]
8764	dipeptidyl peptidase IV inhibitor	1	AV	[433-434]
8765	dipeptidyl peptidase IV inhibitor	2	AY	[164-165],[325-326]
8767	dipeptidyl peptidase IV inhibitor	1	DP	[52-53]
8770	dipeptidyl peptidase IV inhibitor	1	EG	[88-89]
8772	dipeptidyl peptidase IV inhibitor	2	EI	[231-232],[398-399]
8774	dipeptidyl peptidase IV inhibitor	3	ET	[222-223],[438-439],[525-526]
8775	dipeptidyl peptidase IV inhibitor	2	EV	[277-278],[314-315]
8777	dipeptidyl peptidase IV inhibitor	3	EY	[44-45],[75-76],[243-244]
8783	dipeptidyl peptidase IV inhibitor	1	GG	[5-6]
8784	dipeptidyl peptidase IV inhibitor	2	GH	[193-194],[453-454]
8785	dipeptidyl peptidase IV inhibitor	4	GI	[16-17],[89-90],[100-101],[195-196]
8786	dipeptidyl peptidase IV inhibitor	3	GV	[47-48],[79-80],[125-126]
8788	dipeptidyl peptidase IV inhibitor	1	GY	[403-404]
8790	dipeptidyl peptidase IV inhibitor	1	HE	[291-292]

8794	dipeptidyl peptidase IV inhibitor	1	HR	[246-247]
8800	dipeptidyl peptidase IV inhibitor	3	IH	[467-468],[566-567],[596-597]
8801	dipeptidyl peptidase IV inhibitor	1	II	[442-443]
8802	dipeptidyl peptidase IV inhibitor	8	IL	[101-102],[142-143],[196-197],[198-199],[330-331],[443-444],[464-465],[580-581]
8803	dipeptidyl peptidase IV inhibitor	1	IM	[484-485]
8805	dipeptidyl peptidase IV inhibitor	7	IQ	[207-208],[232-233],[240-241],[263-264],[302-303],[426-427],[498-499]
8806	dipeptidyl peptidase IV inhibitor	1	IR	[423-424]
8807	dipeptidyl peptidase IV inhibitor	1	IW	[316-317]
8808	dipeptidyl peptidase IV inhibitor	2	KE	[347-348],[493-494]
8809	dipeptidyl peptidase IV inhibitor	4	KF	[73-74],[297-298],[309-310],[344-345]
8810	dipeptidyl peptidase IV inhibitor	1	KG	[10-11]
8812	dipeptidyl peptidase IV inhibitor	5	KI	[18-19],[267-268],[389-390],[463-464],[579-580]
8813	dipeptidyl peptidase IV inhibitor	2	KK	[110-111],[492-493]
8815	dipeptidyl peptidase IV inhibitor	2	KS	[557-558],[561-562]
8816	dipeptidyl peptidase IV inhibitor	5	KT	[111-112],[128-129],[146-147],[321-322],[541-542]
8817	dipeptidyl peptidase IV inhibitor	6	KV	[22-23],[83-84],[156-157],[289-290],[447-448],[575-576]
8819	dipeptidyl peptidase IV inhibitor	3	KY	[251-252],[349-350],[410-411]
8820	dipeptidyl peptidase IV inhibitor	2	LH	[245-246],[511-512]
8821	dipeptidyl peptidase IV inhibitor	9	LI	[24-25],[197-198],[206-207],[265-266],[301-302],[364-365],[380-381],[384-385],[422-423]
8822	dipeptidyl peptidase IV inhibitor	2	LM	[33-34],[605-606]
8823	dipeptidyl peptidase IV inhibitor	5	LN	[95-96],[102-103],[107-108],[150-151],[279-280]
8824	dipeptidyl peptidase IV inhibitor	5	LT	[60-61],[69-70],[144-145],[355-356],[559-560]
8825	dipeptidyl peptidase IV inhibitor	5	LV	[37-38],[54-55],[227-228],[282-283],[532-533]
8826	dipeptidyl peptidase IV inhibitor	1	ME	[221-222]
8828	dipeptidyl peptidase IV inhibitor	2	MG	[161-162],[530-531]
8830	dipeptidyl peptidase IV inhibitor	2	MI	[407-408],[497-498]
8831	dipeptidyl peptidase IV inhibitor	1	MK	[556-557]

8832	dipeptidyl peptidase IV inhibitor	4	ML	[34-35],[429-430],[517-518],[606-607]
8834	dipeptidyl peptidase IV inhibitor	1	MN	[215-216]
8835	dipeptidyl peptidase IV inhibitor	1	MQ	[118-119]
8836	dipeptidyl peptidase IV inhibitor	1	MR	[477-478]
8837	dipeptidyl peptidase IV inhibitor	2	MV	[93-94],[370-371]
8839	dipeptidyl peptidase IV inhibitor	1	NA	[451-452]
8841	dipeptidyl peptidase IV inhibitor	1	NE	[204-205]
8843	dipeptidyl peptidase IV inhibitor	2	NG	[78-79],[216-217]
8844	dipeptidyl peptidase IV inhibitor	2	NH	[151-152],[280-281]
8845	dipeptidyl peptidase IV inhibitor	2	NL	[178-179],[545-546]
8847	dipeptidyl peptidase IV inhibitor	1	NN	[602-603]
8850	dipeptidyl peptidase IV inhibitor	2	NT	[31-32],[374-375]
8851	dipeptidyl peptidase IV inhibitor	5	NV	[96-97],[103-104],[114-115],[270-271],[286-287]
8853	dipeptidyl peptidase IV inhibitor	1	NY	[186-187]
8854	dipeptidyl peptidase IV inhibitor	1	PF	[211-212]
8857	dipeptidyl peptidase IV inhibitor	7	PI	[71-72],[236-237],[239-240],[262-263],[425-426],[460-461],[466-467]
8858	dipeptidyl peptidase IV inhibitor	1	PK	[50-51]
8859	dipeptidyl peptidase IV inhibitor	3	PM	[369-370],[476-477],[529-530]
8863	dipeptidyl peptidase IV inhibitor	1	PT	[455-456]
8864	dipeptidyl peptidase IV inhibitor	4	PV	[191-192],[394-395],[416-417],[419-420]
8867	dipeptidyl peptidase IV inhibitor	3	QA	[241-242],[272-273],[362-363]
8868	dipeptidyl peptidase IV inhibitor	2	QD	[174-175],[427-428]
8869	dipeptidyl peptidase IV inhibitor	3	QE	[119-120],[154-155],[499-500]
8870	dipeptidyl peptidase IV inhibitor	2	QF	[208-209],[319-320]
8871	dipeptidyl peptidase IV inhibitor	1	QG	[124-125]
8873	dipeptidyl peptidase IV inhibitor	1	QI	[274-275]
8874	dipeptidyl peptidase IV inhibitor	5	QL	[67-68],[106-107],[264-265],[303-304],[504-505]
8875	dipeptidyl peptidase IV inhibitor	3	QN	[113-114],[440-441],[544-545]

8876	dipeptidyl peptidase IV inhibitor	1	QQ	[173-174]
8877	dipeptidyl peptidase IV inhibitor	1	QS	[225-226]
8878	dipeptidyl peptidase IV inhibitor	1	QT	[260-261]
8879	dipeptidyl peptidase IV inhibitor	1	QV	[547-548]
8880	dipeptidyl peptidase IV inhibitor	1	QW	[328-329]
8882	dipeptidyl peptidase IV inhibitor	3	RG	[99-100],[180-181],[247-248]
8884	dipeptidyl peptidase IV inhibitor	1	RI	[141-142]
8886	dipeptidyl peptidase IV inhibitor	2	RL	[584-585],[587-588]
8887	dipeptidyl peptidase IV inhibitor	2	RM	[496-497],[516-517]
8888	dipeptidyl peptidase IV inhibitor	2	RN	[285-286],[488-489]
8891	dipeptidyl peptidase IV inhibitor	1	SF	[550-551]
8892	dipeptidyl peptidase IV inhibitor	1	SH	[554-555]
8894	dipeptidyl peptidase IV inhibitor	5	SK	[21-22],[176-177],[386-387],[409-410],[593-594]
8895	dipeptidyl peptidase IV inhibitor	2	SV	[367-368],[569-570]
8897	dipeptidyl peptidase IV inhibitor	2	SY	[184-185],[552-553]
8899	dipeptidyl peptidase IV inhibitor	4	TE	[166-167],[256-257],[375-376],[542-543]
8903	dipeptidyl peptidase IV inhibitor	2	TI	[356-357],[535-536]
8904	dipeptidyl peptidase IV inhibitor	5	TK	[58-59],[145-146],[223-224],[462-463],[560-561]
8905	dipeptidyl peptidase IV inhibitor	3	TL	[1-2],[32-33],[129-130]
8906	dipeptidyl peptidase IV inhibitor	2	TM	[92-93],[406-407]
8907	dipeptidyl peptidase IV inhibitor	1	TN	[269-270]
8908	dipeptidyl peptidase IV inhibitor	2	TQ	[112-113],[439-440]
8909	dipeptidyl peptidase IV inhibitor	4	TR	[147-148],[284-285],[322-323],[526-527]
8910	dipeptidyl peptidase IV inhibitor	2	TS	[183-184],[456-457]
8911	dipeptidyl peptidase IV inhibitor	3	TT	[61-62],[534-535],[563-564]
8912	dipeptidyl peptidase IV inhibitor	1	TV	[202-203]
8916	dipeptidyl peptidase IV inhibitor	5	VE	[38-39],[276-277],[287-288],[391-392],[420-421]
8917	dipeptidyl peptidase IV inhibitor	2	VF	[84-85],[228-229]

8918	dipeptidyl peptidase IV inhibitor	2	VG	[81-82],[192-193]
8919	dipeptidyl peptidase IV inhibitor	2	VH	[290-291],[479-480]
8920	dipeptidyl peptidase IV inhibitor	2	VI	[157-158],[315-316]
8921	dipeptidyl peptidase IV inhibitor	1	VK	[540-541]
8922	dipeptidyl peptidase IV inhibitor	9	VL	[23-24],[94-95],[104-105],[188-189],[278-279],[395-396],[400-401],[448-449],[522-523]
8924	dipeptidyl peptidase IV inhibitor	1	VN	[203-204]
8925	dipeptidyl peptidase IV inhibitor	1	VQ	[271-272]
8927	dipeptidyl peptidase IV inhibitor	3	VT	[283-284],[371-372],[533-534]
8929	dipeptidyl peptidase IV inhibitor	3	VY	[48-49],[97-98],[115-116]
8932	dipeptidyl peptidase IV inhibitor	2	YA	[76-77],[131-132]
8934	dipeptidyl peptidase IV inhibitor	2	YE	[116-117],[252-253]
8936	dipeptidyl peptidase IV inhibitor	1	YG	[404-405]
8940	dipeptidyl peptidase IV inhibitor	2	YL	[244-245],[350-351]
8941	dipeptidyl peptidase IV inhibitor	1	YM	[506-507]
8942	dipeptidyl peptidase IV inhibitor	2	YN	[185-186],[601-602]
8944	dipeptidyl peptidase IV inhibitor	2	YR	[98-99],[326-327]
8945	dipeptidyl peptidase IV inhibitor	2	YS	[45-46],[553-554]
8946	dipeptidyl peptidase IV inhibitor	1	YV	[187-188]
9334	dipeptidyl peptidase IV inhibitor	1	VAAA	[570-573]
